# Supplementary material for: Long-Term Antibacterial Film Nanocomposite Incorporated with Patchouli Essential Oil Prepared by Supercritical CO2 Cyclic Impregnation for Wound Dressing
Source: Molecules. 2021 Aug 18;26(16):5005. doi: 10.3390/molecules26165005 (PMC8400993; doi:10.3390/molecules26165005)
Supplement: Supplementary file 1 [file molecules-26-05005-s001.zip › molecules-1306555-supplementary.pdf]

Supplementary Materials

Long-Term Antibacterial Film Nanocomposite Incorporated with Patchouli Essential Oil Prepared by Supercritical CO_2 Cyclic Impregnation for Wound Dressing

Jingfu Jia [1,†], Shulei Duan [2,†], Xue Zhou [1], Lifang Sun [1], Chengyuan Qin [1], Ming Li [2,*] and Fahuan Ge [1,*]

[1] School of Pharmaceutical Sciences, Sun Yat-sen University, Guangzhou 510006, China; jiajingfu@mail.sysu.edu.cn (J.J.); zhouxue9@mail.sysu.edu.cn (X.Z.); slf04296923@163.com (L.S.); qinchy8@mail2.sysu.edu.cn (C.Q.)

[2] School of Traditional Chinese Medicine, Guangdong Pharmaceutical University, Guangzhou 510006, China; 17854223645@163.com

* Correspondence: gefahuan@mail.sysu.edu.cn (F.G.); 13539843803@163.com (M.L.); Tel.: +86-20-3909-9733 (F.G.); +86-20-3935-2182 (M.L.)

† The authors contributed equally to this work.

S1. Preparation of mesoporous silica nanoparticles

The mesoporous silica nanoparticles MCM-41 were prepared using the method described previously. Typically, 0.4 mol CTAB and 0.062 mol DEA was mixed in 165 mL water and stirred at 350 r/min under 95 °C, then 1 mol TEOS was dropwise added within 45 min. After reacting for another 1 h, the white emulsion was centrifuged and washed with water and methanol several times. Finally, the obtained particles were soaked in ethanol/HCl (8/1, *v*/*v*) at 60 °C for 24 h to remove the surfactant template, resulting in CTAB free MCM-41.

S2. Analytical methods used

HPLC method

The amount of PEO was calculated based on patchoulenone by using a high-performance liquid chromatography system (HPLC, UltiMate 300, Thermo Fisher Scientific Ltd., America). During the determination, a Sharpsil-HC18 chromatography column (250 L × 4.6 mm I.D., S-5 μm 100A) was used, with acetonitrile/ 0.2% phosphoric acid solution (65:35) as mobile phase. The samples were detected by UV at 310 nm, the temperature was 30 °C, and the flow rate was 0.8 mL/min.

GC-MS method

GC-MS analysis was achieved using an ISQ 7000 instrument (Thermo Fisher Scientific, USA). Patchouli oil were separated on a 30 m × 0.25 mm i.d. capillary column. The column temperature was at 80 °C for injection, held for 5 min. Temperature program began at 10 °C/min to 110 °C, then at 3 °C/min to 120 °C and held for 10 min. Furthermore, the temperature was increased at 2 °C/min to 134 °C, at 1 °C/min to 143 °C and held for 5 min, then at 5 °C/min to 240 °C, finally at 20 °C/min to 280 °C and held for 5 min. Split injection (2μL) was conducted with a split ratio of 1:10 and helium was used as carrier gas of 1.0 mL/min flow-rate. The spectrometers were operated in electron-impact (EI) mode, the scan range was 25–500 amu, the ionization energy was 70 eV, and the scan rate was 0.204 s per scan. The inlet, ionization source temperatures were 230 °C and 250 °C, respectively.

S3. Experiments carried out for preparing PEO-MSNs and investigating the effects of operating parameters on drugload

No.	Pressure (MPa)	Circle index	Loading time (min)	Drugload (%)
1	12	1	60	19.9 ± 0.3
2	15	1	60	28.5 ± 0.3
3	18	1	60	28.2 ± 0.6
4	21	1	60	28.9 ± 0.8
5	12	1	30	9.3 ± 0.5
6	12	1	90	27.8 ± 0.6
7	12	1	120	28.5 ± 0.4
8	15	1	30	24.3 ± 0.7
9	15	1	90	28.0 ± 0.6
10	15	1	120	29.2 ± 0.2
11	15	2	60	37.0 ± 0.4
12	15	3	60	41.8 ± 0.2
13	15	4	60	43.7 ± 0.3
14	15	5	60	43.8 ± 0.5
